# The economic value of personal protective equipment for healthcare workers

**DOI:** 10.1371/journal.pgph.0002043

**Published:** 2023-06-22

**Authors:** Theodore Bolas, Kalin Werner, Sarah Alkenbrack, Manuela Villar Uribe, Mengxiao Wang, Nicholas Risko

**Affiliations:** 1 Department of International Health, Johns Hopkins Bloomberg School of Public Health, Baltimore, Maryland, United States of America; 2 Institute for Health & Aging, University of California, San Francisco, San Francisco, California, United States of America; 3 Division of Emergency Medicine, University of Cape Town, Cape Town, South Africa; 4 World Bank, Health, Nutrition and Population Global Practice, Washington DC, United States of America; 5 Johns Hopkins School of Medicine, Baltimore, Maryland, United States of America; University of the Western Cape, SOUTH AFRICA

## Abstract

In this paper, we examine the cost effectiveness of investment in personal protective equipment (PPE) for protecting health care workers (HCWs) against two infectious diseases: Ebola virus and methicillin-resistant *Staphylococcus aureus* (MRSA). This builds on similar work published for COVID-19 in 2020. We developed two separate decision-analytic models using a payer perspective to compare the costs and effects of multiple PPE use scenarios for protection of HCW against Ebola and MRSA. Bayesian multivariate sensitivity analyses were used to consider the uncertainty surrounding all key parameters for both diseases. We estimate the cost to provide adequate PPE for a HCW encounter with an Ebola patient is $13.04, which is associated with a 97% risk reduction in infections. The mean incremental cost-effectiveness ratio (ICER) is $3.98 per disability-adjusted life year (DALY) averted. Because of lowered infection and disability rates, this investment is estimated to save $132.27 in averted health systems costs, a financial ROI of 1,014%. For MRSA, the cost of adequate PPE for one HCW encounter is $0.88, which is associated with a 53% risk reduction in infections. The mean ICER is $362.14 per DALY averted. This investment is estimated to save $20.18 in averted health systems costs, a financial ROI of 2,294%. In terms of total health savings per death averted, investing in adequate PPE is the dominant strategy for Ebola and MRSA, suggesting that it is both more costly and less clinically optimal to not fully invest in PPE for these diseases. There are many compelling reasons to invest in PPE to protect HCWs. This analysis examines the economic case, building on previous evidence that protecting HCWs with PPE is cost-effective for COVD-19. Ebola and MRSA scenarios were selected to allow assessment of both endemic and epidemic infectious diseases. While PPE is cost-effective for both conditions, compared to our analysis for COVID-19, PPE is relatively more cost-effective for Ebola and relatively less so for MRSA. Further research is needed to assess shortfalls in the PPE supply chain identified during the COVID-19 pandemic to ensure an efficient and resilient supply in the face of future pandemics.

## Introduction

Personal protective equipment (PPE) is a critical tool for protecting healthcare workers (HCWs), containing outbreaks, and ensuring continuity of health services during pandemics. As a result of the ongoing coronavirus disease 2019 (COVID-19) pandemic, a global spotlight has focused on the availability and usage of PPE. It has become evident that HCWs are often unable to access a reliable supply of high-quality PPE to ensure appropriate protection at work, jeopardizing the safety of themselves and their families and resulting in a missed opportunity to contain the spread of disease. Not surprisingly, inadequate access to PPE is significantly worse in low-income settings that have poorer funding of health systems overall [1].

Scholarly work has begun to emerge that examines and quantifies PPE shortages, outlines challenges with quality and procurement, and diagnoses bottlenecks in deployment and utilization of PPE. At the start of the 2020 pandemic, a global collaboration, Access to COVID-19 Tools Accelerator (ACT-A), reported on issues with the PPE supply chain and provided a roadmap for action [1]. The importance of strengthening the PPE supply chain was validated in late 2020, when research found that if an investment of $9.6 billion USD was provided to protect all HCWs in low- and middle- income countries (LMICs), the societal return on investment (ROI) would be $755.2 billion USD, or a 7,932% return [2]. Country specific estimates have also been produced, which noted similarly strong returns on investment [3, 4].

Despite increased knowledge on the cost-effectiveness of PPE in the setting of COVID-19, less attention has been paid to the impact of PPE investments for other infectious diseases. In this study, we use two other conditions—Ebola and Methicillin-resistant *Staphylococcus aureus* (MRSA)—to explore the cost-effectiveness of PPE in a broader setting. These two diseases present an opportunity to examine the cost-effectiveness of PPE in markedly different epidemiologic scenarios. This is important because needs in the health sector often exceed available resources. Thus, policy makers need evidence regarding the impact of their investments as they set priorities that will inform budgets and plans.

Ebola is a highly infectious viral disease, which spreads via contact with bodily fluids and is fatal in the majority of cases [5, 6]. Although the first outbreak was recorded in the Democratic Republic of the Congo, in 1976, the largest outbreak by far occurred in West Africa in 2014, primarily in Guinea, Liberia, and Sierra Leone [7]. These countries had a shared context of weak health systems, low incomes, and few HCWs [8]. This outbreak disease killed 507 HCWs, creating significant strain on their already weakened health systems [9]. As of January 2023 there is one active major Ebola epidemic in Uganda which has killed 142 people and 7 health care workers [10]. However, transmission is preventable, with infection rates being 28 times lower in conditions with adequate access to necessary PPE [11, 12].

Whereas Ebola is usually present only in rare LMIC based outbreaks and has a long list of required PPE for effective protection, MRSA is highly prevalent globally and only requires basic contact precautions for effective protection [13, 14]. MRSA, a multi-drug resistant S*taphylococcus* strain is a disease of significant concern, with MRSA bacteremia in critically ill patients having a 63.8% mortality rate, compared to 23.7% for methicillin-susceptible strains [15]. MRSA started primarily as a hospital-acquired infection but has since expanded to community transmission, with between 0.9–1.5% of Americans exhibiting nasal colonization [16]. Because of its high mortality rate and relative ease of prevention, providing PPE for MRSA is critical in preventing hospital-acquired infections and deaths.

This paper uses an economic lens to evaluate the cost-effectiveness and the financial return on investment for providing HCWs with adequate PPE for both Ebola and MRSA, using a health systems payer perspective. In other words, it looks at the economic and health benefits associated with an increased investment in PPE and compares them to associated economic costs of this investment. The aim is to inform decisions on policy, strategy, and financing of PPE to protect HCWs both in epidemic settings and daily work beyond the context of COVID-19.

## Methods

We developed two separate decision-analytic models to compare the costs and effects of two PPE use scenarios for protection of HCW against [1] Ebola and [2] MRSA, following standard guidelines for cost-effectiveness analyses and adhering to Consolidated Health Economic Evaluation Reporting Standards (CHEERS) [17, 18]. The analysis was conducted using the HeRo3 Software [19].

For each disease, a base case cohort in which adequate PPE access maintains a low rate of HCW infection was compared to a scenario where inadequate PPE access leads to higher rates of HCW infection. The base case epidemiological parameters, such as infection rate, mortality, and PPE efficacy came from population averages, with MRSA parameters coming primarily from middle-income nations and Ebola parameters coming from low-income sub-Saharan African populations. A decision tree was created for each disease that calculated the probability of falling ill given PPE status, and the resulting odds of death, disability, or recovery as well as the costs of treatment associated with these outcomes. These cohorts were then entered into a lifetime horizon Markov model where they accumulated DALYs and chronic disability costs until their death or the age of 85, at which point mortality was assumed to be 100%. Costs and outcomes were both discounted at 3% annually. Discounting is used in cost-effectiveness analysis studies in order to properly weight immediate and long term benefits and costs, based on the assumption that future benefits and costs are less valuable and less costly than those incurred in the present [20]. The appropriate discount rate can vary based on types of outcomes and societal values; however, most global health studies use a rate of 3% annually, which was chosen for this study in order to ensure comparability across papers [21].

We took a payer perspective, including all costs to the health care system such as cost of treatment and cost of prolonged disability care, in our model. To estimate PPE resource use and costs, we utilized the WHO COVID-19 Essential Supplies Forecasting Tool (ESFT) [18]. The ESFT is designed to help governments and other stakeholders estimate essential supply requirements. Ideal PPE availability is consistent with WHO best practice guidelines. PPE costs in ESFT are intended to directly inform procurement and reflect competitive prices in the global market.

The main health outcomes of interest were: [1] Disability Adjusted Life Years (DALY) averted, [2] HCW infections averted, and [3] HCW deaths averted. We tracked the costs associated with each outcome and calculated the incremental cost-effectiveness ratio (ICER) expressed as the ratio of cost per unit of effect, for both the PPE costs and the total health systems costs. A Return on Investment (ROI) analysis was also performed by calculating the health systems savings per dollar invested in PPE.

Finally, we performed a Bayesian multivariate sensitivity analysis to consider the uncertainty surrounding all key parameters for both diseases. A Monte Carlo simulation randomly re-sampled iteratively across the input distributions for each model parameter. Where available, input distributions were based on mean values from the literature and their associated 95% confidence intervals. Beta distributions were used for sampling for probability variables and gamma distributions for cost variables. The design of the sensitivity analysis, including simulation runs and the distributions selected, was based on international standards for cost-effectiveness analysis [17, 18]. We present these results as cost-effectiveness planes.

### Ebola model

**Fig 1:**
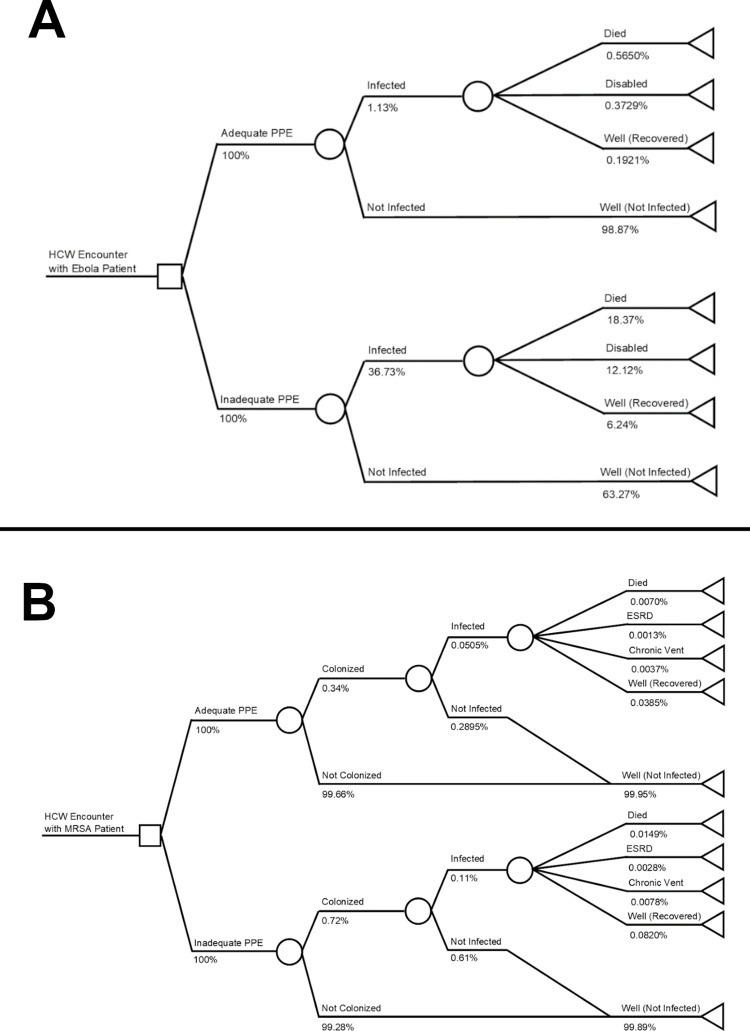
a. Ebola Decision Tree. b. MRSA Decision Tree Model.

For Ebola, adequate PPE sharply lowers the infection rate based on data from spillover cases into the US and Spain [11, 12]. These data were chosen as there were no community cases in these countries, and thus all cases can be traced to a single source. The impact of this is shown in the decision tree (Fig 1A.). Adequate PPE consists of: a protective apron, gown, boots, a scrub top and bottom, a face shield, respirator, two pairs of gloves, and soap [22].

Treatment costs were taken from a review of the costs of care in Liberia, Guinea, and Sierra Leone during the 2013–2016 outbreak in those countries [23]. Although these costs may be different due to the epidemic conditions, they are likely consistent with other outbreaks in the region. Based on data from Sierra Leone, the cost of illness was varied between survivors and non-survivors, where survivors required significantly more hospitalized care than those who succumbed to illness [24]. The literature surrounding the long-term health impacts of Ebola survivors is limited, however, we found published estimates on morbidity from Sierra Leone that estimate 66% of survivors suffer long-term disability [25]. We used the DALY weight for Post-Ebola Chronic Fatigue from the 2019 Global Burden of Disease Study to estimate the total burden [26]. For survivors with chronic disability, long-term care costs were included.

Once per-encounter probabilities were calculated, each cohort was entered into a Markov model. We used this to model the impact of moving between living health states (never-infected, recovered, or disabled) and to death. All groups faced the average sub-Saharan African mortality rate for their age group and sex, and all mortality rates were set to 1 at age 85, as this is the last age with data in the life tables [27]. DALYs were accumulated annually in their respective groups ranging from 0 to 1 depending on health state. All variables used in the model are listed in Table 1.

**Table 1:** Ebola model parameter range and distribution.

Parameter	Value	Distribution	Source
***Clinical Parameters***			
Infection Rate with Inadequate PPE	0.3673 (0.344–0.391)	Beta	[28]
Infection Rate with Adequate PPE	0.0113 (0.0105–0.0121)	Beta	[11, 12]
Case Fatality Rate	0.5 (0.421–1)	Beta	[29]
Disability Rate	0.33 (0.2805–0.38)	Beta	[25]
Mortality Rate if Healthy	From African Life Tables	-	[27]
***Costs (USD 2022)***			
Apron	$0.20 (0.17–0.23)	Gamma	[30]
Boots	$4.60 (3.91–5.29)	Gamma	[30]
Soap	$0.02 (0.0153–0.0207)	Gamma	[30]
Gown	$0.80 (0.68–0.92)	Gamma	[30]
Scrub Top	$2.60 (2.21–2.99)	Gamma	[30]
Scrub Bottom	$2.60 (2.21–2.99)	Gamma	[30]
Face Shield	$0.60 (0.51–0.69)	Gamma	[30]
Gloves x2	$0.12 (0.102–0.14)	Gamma	[30]
Respirator	$1.50 (1.275–1.73)	Gamma	[30]
Cost of Ebola Treatment (Survive)	$596.91 (404.91–789.91)	Gamma	[23]
Cost of Ebola Treatment (Death)	$211.91 (143.91–279.91)	Gamma	[23]
Cost of disability treatment	$21 (14–28)	Gamma	Expert Assumption
***DALY Weights***			
Fatigue	0.291 (0.148–0.308)	Beta	[26]

### MRSA model

For MRSA, PPE is considered adequate when healthcare workers had access to a gown, a pair of gloves, and adequate soap under the guidelines for the US Centers for Disease Control [14]. Our decision model for MRSA assumes effective PPE can reduce the risk of colonization of MRSA, and that a small number of MRSA colonizations will result in fulminant infection, based on literature values. To account for this, we first apply the risk of colonization with adequate or inadequate PPE per encounter, and then subsequently apply the risk of infection given one is colonized [31]. Most infections result in a full recovery, although a small proportion of those infected will suffer critical illness, death, or long-term disabilities such as chronic ventilator use or end stage renal disease (ESRD) [32, 33]. This is shown in the decision tree for MRSA, Fig 1B.

PPE costs were calculated using the WHO ESFT and we assumed inadequate PPE required no new investment into PPE supplies. Treatment costs were available from several countries; however, we chose costs from a middle-income setting to be more generalizable to the global context [34]. Once per-encounter probabilities were calculated, each cohort was entered into a Markov model. This model allowed patients to move from their respective health states (never-infected, recovered, ESRD, and chronic ventilator use) to death. Age-adjusted mortality rates were applied, including specific rates for patients with ESRD and an annual mortality rate for ventilator dependence [27, 35, 36]. All mortality rates were consistent with data in the global life tables [27]. Annual chronic care costs were applied to the ESRD and chronic ventilator use group. Burden of disability was derived from the WHO-CHOICE DALY weights that closest resembled the two most common long-term morbidities from severe MRSA infection, including [1] end-stage renal disease on dialysis without anemia due to other and unspecified cause and [2] severe chronic obstructive pulmonary disease without heart failure [26]. These DALY weights were accumulated annually in their respective Markov groups, while those without disability accumulated 0 DALYs and those who were dead accumulated 1 DALY annually. Parameter values can be found in Table 2.

**Table 2:** MRSA model parameter range and distribution.

Parameter	Value	Distribution	Source
***Clinical Parameters***			
Effectiveness of PPE in Preventing Colonization	0.47 (0.43–0.51)	Beta	[37]
Colonization Probability per Admission	0.25 (0.2125,0.2875)	Beta	[38]
Encounters per Admission	40 (30–50)	Beta	Expert Assumption
Colonization Probability per Encounter	0.00716	Beta	Derived Value
Infection Rate per Colonization	0.15 (0.1–0.3)	Beta	[31]
Case Fatality Rate	0.14 (0.048–0.229)	Beta	[33]
Chronic Ventilator Dependence Rate	0.073 (0.06025–0.08395)	Beta	[32]
Dialysis Dependent End Stage Renal Disease Rate	0.026 (0.0221–0.0299)	Beta	[32]
Mortality Rate if Healthy	From Global Life Tables	-	[27]
Mortality Rate with ESRD	From ESRD Life Tables	-	[36]
Mortality Rate with Chronic Ventilator Use	0.3186 (0.1706,0.4159)	Beta	[35]
***Costs***			
Soap	$0.02 (0.0153–0.0207)	Gamma	[30]
Gown	$0.8 (0.68–0.92)	Gamma	[30]
Gloves	$0.06 (0.051–0.069)	Gamma	[30]
Cost of MRSA Treatment (Survivor)	$11,178 (10,357–12,155)	Gamma	[34]
Cost of MRSA Treatment (Non-Survivor)	$18,069 (12,965–21,375)	Gamma	[34]
Cost of chronic ventilator care (per patient per year)	$63,300 (27,492–122,172)	Gamma	[39]
Cost of ESRD care (per patient per year)	$38,580 (18875–43709)	Gamma	[40]
***DALY Weights***			
Chronic Ventilator Use	0.8 (0.7–0.9)	Beta	[26]
End Stage Renal Disease	0.571(.0398–0.725)	Beta	[26]

## Results

The cost to provide adequate PPE for a single HCW encounter with an Ebola patient is estimated to be $13.04. This investment is associated with a 97% reduction in the probability of infections and resulting disabilities, and deaths, and a total of 3.28 DALYs averted per protected HCW. The mean incremental cost-effectiveness ratio (ICER) from the payer perspective is $3.98 per DALY averted. Because of lowered infection and disability rates, every $13.04 invested in PPE is estimated to avert $132.27 in health systems costs—a financial ROI of 1014%, as shown in Table 3. When considering the additional savings noted from the health sector perspective, the investment in PPE becomes cost-saving and improves health outcomes, making it the dominant strategy.

**Table 3:** Cost-effectiveness and return on investment for adequate PPE investment for protection against Ebola per HCW encounter.

	**Outcomes**				**Costs**	
	**HCW Cases**		**HCW Deaths**	**HCW DALYs**	**PPE Costs**	**Total Health Systems Costs**
**Incremental Change**	-0.356		-0.178	-3.279814	$13.04	-$132.27
**Inadequate PPE**	0.3673		0.18365	15.00437	$0.00	$148.51
**Adequate PPE**	0.0113		0.00565	11.72456	$13.04	$1.62
**Cost-Effectiveness Ratios**						
**PPE Cost per Case Averted**	**PPE Cost per Death Averted**	**PPE Cost per DALY Averted**	**Total Costs per Case Averted**	**Total Costs per Death Averted**	**Total Costs per DALY Averted**	**Health Systems Savings per dollar invested in PPE**
$36.62	$73.25	$3.98	Strongly Dominant	Strongly Dominant	Strongly Dominant	$10.14

We estimate that the cost of adequate PPE for one HCW encounter with a MRSA patient is $0.88. This investment is associated with a 53% reduction in the probability of infections and resulting disabilities and deaths, with a total of 0.00243 DALYs averted per HCW-patient encounter. The mean ICER from the payer perspective is $362.14 per DALY averted. Due to lowering of infection and disability rates, every dollar invested in PPE is estimated to avert $20.18 in health systems costs - a financial ROI of 2,294%, as shown in Table 4. When considering these additional averted costs from the health sector perspective, the investment in PPE becomes cost-saving and improves health outcomes, making it the dominant strategy.

**Table 4:** Cost-effectiveness and return on investment for adequate PPE investment for protection against MRSA per HCW encounter.

**Outcomes**					**Costs**	
	**HCW Cases**	**HCW Deaths**		**HCW DALYs**	**PPE Costs**	**Total Health Systems Costs**
**Incremental Change**	-0.000491	-0.000079		-0.002430	$0.88	-$20.18
**Inadequate PPE**	0.000926	0.000149		8.431302	$0.00	$39.74
**Adequate PPE**	0.000435	0.000070		8.428872	$0.88	$19.56
**Cost-Effectiveness Ratios**						
**PPE Cost per Case Averted**	**PPE Cost per Death Averted**	**PPE Cost per DALY Averted**	**Total Costs per Case Averted**	**Total Costs per Death Averted**	**Total Costs per DALY Averted**	**Health Systems Savings per dollar invested in PPE**
$1,792.26	$11,139.24	$362.14	Strongly Dominant	Strongly Dominant	Strongly Dominant	$22.94

**Fig 2:**
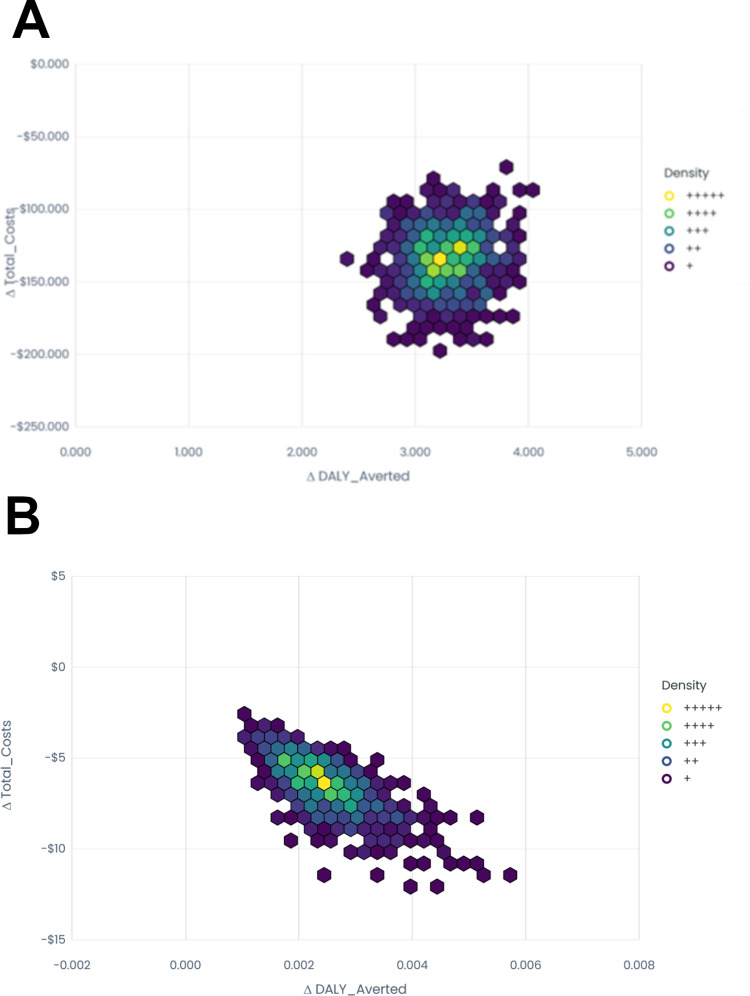
a. Ebola cost-effectiveness plane hexbin plot. b. MRSA cost-effectiveness plane hexbin plot.

Fig 2A and 2B contain cost-effectiveness plane hexbin plots representing the relationship between DALYs averted and health systems costs for the Ebola and MRSA scenarios respectively.

The results of the probabilistic sensitivity analysis showed that, for both Ebola and MRSA, DALYs averted were positive and total costs were negative across all simulations. This suggests robustness in our findings that use of adequate PPE is the dominant strategy over inadequate PPE.

## Discussion

This paper analyzes the cost-effectiveness and financial ROI of providing HCWs with adequate PPE for both Ebola and MRSA, with the aim of informing decision makers in this area beyond the context of COVID-19. Our findings demonstrate that investments in PPE for the two conditions yield strong returns in both health and future cost-savings. Literature on PPE for COVID-19 has already supported this conclusion [2, 3]. Taken together, this evidence supports a broad economic case for increasing investments to improve PPE access in health systems across the globe.

There are several limitations to this research that are worth noting. Available data relating to costs of treating MRSA mostly reflect high-income settings. However, we were able to identify and utilize data from China that should be more generalizable to global settings. Overall, results for MRSA may be less accurate for lower-income countries. With regards to Ebola, given that the majority of outbreaks have occurred in west and sub-Saharan Africa, our data and results are most reflective of those settings. Higher costs of care in a given country would be associated with a higher level of health systems savings from PPE investment, while higher quality of other infection control practices may lower the relative benefit of PPE. Fortunately, the price of PPE itself is fairly normalized across the globe and tends not to vary significantly by region [41].

We used a highly conservative approach by not taking into account the societal benefits of averting death and disability amongst HCWs, which are substantial, particularly in times of pandemics and HCW scarcity. While the loss of any HCW weakens the overall health system and its ability to continue providing services, the effect is amplified in low resource settings that are already undergoing a crisis of human resources in health [42]. The training of replacement HCWs is costly and requires significant time. Furthermore, our model does not consider or attempt to quantify the downstream effects of HCW death and disability on the health and productivity of countries. As such, our calculations likely underestimate the true social and health return on investment of protecting healthcare workers, particularly in LMICs.

In order to successfully model future scenarios related to PPE cost-effectiveness, assumptions were made around the stability of supply, ease of access, and appropriateness of use, as detailed below. The model assumes that an investment in PPE at the health system level will result in availability and correct use at the bedside. Although limited supply of PPE does occur in the real world, this paper shows the large benefits associated with sufficient access to PPE, as an argument for increased investment. We also assumed that rationing of PPE to extend its use beyond manufacturer recommendations would not be occurring. Despite reports of this happening widely during the COVID-19 pandemic, it represents an inappropriate method of cost-saving and resource conservation. While our modeling assumptions depart from these real-world distortions, it is unclear how they would influence the results if included, and we believe excluding reuse to be the approach of least bias. Furthermore, we attempted to account for the uncertainty in our parameters within our sensitivity analysis.

On its own, cost-effectiveness analysis is limited in informing what is actually budgeted by countries. Resources are scarce, priorities are not always driven by evidence, and countries cannot always afford to have a full supply of PPE. In most LMICs, additional resources would be needed to secure a reliable PPE supply. This could come in the form of domestic resources, either new or reallocated from other areas of less productive spending, or it could be in the form of development assistance aligned to country priorities. There is also a need to explore opportunities for shaping markets to increase affordable, climate-friendly supply options, given the environmental effects that go along with PPE use and waste disposal. Additionally, rationing of PPE supply tends to coincide with widespread challenges of procurement processes and difficulties ensuring adequate deployment and utilization at the point of service delivery. Strengthening the resilience of PPE supply chains is an important component of pandemic prevention, preparedness, and response, as it will protect HCWs, reduce illness, prevent disease outbreaks, and mitigate the disruption of essential services.

## Conclusion

PPE is a critical tool for protecting HCWs, containing outbreaks, and ensuring continuity of health services during pandemics. However, too often, HCWs in low- and lower-middle-income countries are not able to access a reliable, adequate supply of PPE. This study demonstrates the cost-effectiveness of providing an adequate supply of PPE for two diseases that represent epidemic (Ebola) and endemic (MRSA) disease scenarios. There are many compelling reasons to protect HCWs, and this evidence highlights one of those reasons and augments the growing body of literature on this topic and is important for informing priority setting in the health sector.

## Supporting information

S1 Data Five supporting tables. **MRSA Clinical Parameters:** A list of all clinical values used for our MRSA model including mortality, PPE Effectiveness, and other epidemiological parameters associated with the disease; **Ebolas Clinical Parameters:** A list of all clinical values used for our Ebola model including mortality, PPE Effectiveness, and other epidemiological parameters associated with the disease; **PPE Costs:** A list of all costs for all PPE according to the WHO nCoV tools forecast. **MRSA Costs:** A list of all costs associated with MRSA treatment and the treatment of MRSA-related disability; **Ebola Costs:** A list of all costs associated with MRSA treatment and the treatment of MRSA-related disability. All tables have the same 5 columns: **Parameter:** An explanation of the parameter’s use in the model; **Value:** The point value used in the non-probabilistic model; **Standard Deviation:** The standard deviation used in the probabilistic model; **Distribution:** The type of probability distribution used; **Source:** The source from which the data was drawn.
(XLSX)
